# Inoca and Its Diagnosis by Microvascular Study, A Case Report

**DOI:** 10.3390/reports9030196

**Published:** 2026-06-23

**Authors:** Bomonyo Fente, Ahmad El-Said, Hilda Yuson, Gavin Galasko

**Affiliations:** Blackpool Victoria Hospital, Blackpool FY3 8NP, UK; ahmad.elsaid2@nhs.net (A.E.-S.); hilda.yuson@nhs.net (H.Y.); dr.galasko@nhs.net (G.G.)

**Keywords:** ischaemia with non-obstructive coronary arteries (INOCA), coronary microvascular dysfunction (CMD), coronary artery disease (CAD), invasive coronary angiography, intracoronary nitrate

## Abstract

**Background and Clinical Significance**: Ischaemia with non-obstructive coronary arteries (INOCA) has attained more recognition in recent decades. These patients may present with typical cardiac sounding chest pain but have no evidence of obstructed coronary arteries on coronary angiography. This presents a challenge to clinicians in terms of diagnosis and management. Coronary microvascular dysfunction (CMD), or coronary spasm (whether epicardial or microvascular) may be the cause of their presentation, and they usually require further invasive investigations of their coronary microvascular circulation to determine the cause. **Case Presentation**: This case involves a male patient in his 60s presenting with recurrent nocturnal chest pain, clinical and ECG evidence of ischaemia, and diagnostic findings from invasive coronary angiography and a microvascular study. These findings confirmed an absence of obstructive coronary artery disease (CAD) but demonstrated significant microvascular dysfunction, consistent with a diagnosis of microvascular angina according to the COVADIS criteria, as well as epicardial coronary artery spasm leading to complete vessel closure. This case highlights the clinical and diagnostic complexities of microvascular angina and coronary artery spasm. It also emphasises the importance of advanced diagnostic testing in confirming this challenging diagnosis. This case was interesting due to the patient having a final diagnosis of microvascular angina and coronary artery spasm at the same time. This case also demonstrates how 300 mcg of intracoronary nitrate was given to dilate a vessel in coronary spasm with positive effect. This finding was supportive of the final diagnosis given the clinical context of this patient. **Conclusions**: This case report demonstrates the diagnostic steps, from symptom assessment through to angiography and microvascular testing and would add to the existing knowledge of INOCA and aid in the understanding and management of these patients especially in centres where acetylcholine testing to confirm inducible epicardial coronary spasm is not available, like it was not in our centre (Blackpool Victoria Hospital).

## 1. Background and Clinical Significance

Ischaemia with non-obstructive coronary arteries (INOCA) has attained more recognition in recent decades. Patients may present with typical cardiac sounding chest pain but have no evidence of obstructed coronary arteries on coronary angiography. This presents a challenge to clinicians in terms of diagnosis and management. Coronary microvascular dysfunction (CMD), or coronary spasm (whether epicardial or microvascular) may be the cause of their presentation.

We present a male in his 60s presenting with recurrent nocturnal chest pain, clinical and ECG evidence of ischaemia, and diagnostic findings from invasive coronary angiography and a microvascular study. These findings confirmed an absence of obstructive coronary artery disease (CAD) but demonstrated significant microvascular dysfunction, consistent with a diagnosis of microvascular angina and epicardial coronary artery spasm according to the COVADIS criteria. We highlight the clinical and diagnostic complexities of microvascular angina and coronary artery spasm, emphasizing the importance of advanced diagnostic testing in confirming this diagnosis.

Chest pain due to coronary ischaemia is commonly seen by cardiologists, with further investigations frequently showing narrowed epicardial coronary arteries as the cause. In recent years however, it has become increasingly apparent that many patients presenting with both typical and atypical chest pain symptoms have no obstructive epicardial coronary artery disease but do have abnormalities in the anatomy and physiology of their coronary microcirculation as the cause of their symptoms. Many patients also have episodes of coronary artery spasm as the cause of their symptoms, whether epicardial or microvascular, with their vessels episodically constricting leading to symptoms of chest pain.

This is known as ischaemia with non-obstructive coronary arteries (INOCA). It describes patients who present with angiographic evidence of ischaemia, but no obstructive coronary disease (CAD) at coronary angiography [1]. This is a prevalent health issue with as many as 70% of all patients undergoing coronary angiography due to angina and myocardial ischaemia having no obstructed coronary arteries despite having evidence of ischaemia [2]. While not having obstructed coronary arteries, the majority of patients with INOCA have been found to have abnormal coronary microvascular dysfunction (microvascular angina) and/or epicardial and/or microvascular coronary vascular dysfunction (coronary artery spasm) [1]. These are now recognised as additional pathophysiological mechanisms of ischaemic heart disease [3]. Studies have shown that patients presenting with INCOA despite having non-obstructive coronary artery disease are still associated with adverse cardiovascular outcomes, recurrent hospitalisations, and poorer quality of life [1] making it an important and distressing reality for patients. Specific medications have also been shown to improve symptoms, once the diagnosis has been made. This is the case of a patient found to have both microvascular angina and coronary artery spasm as the cause of his symptoms, in the absence of obstructive coronary artery disease.

## 2. Case Presentation

A male in his 60s with recurrent nocturnal central chest pain was referred to the cardiology clinic. His symptoms began shortly after his last hospital admission three years ago for a non-ST-elevation myocardial infarction (NSTEMI), and they had persisted despite ongoing medical therapy. The chest pain episodes primarily occurred at night and were alleviated by glyceryl trinitrate spray. He described the pain as “heart-burn like” accompanied by a sour taste—not a usual characteristic for cardiac chest pain. During the day, he occasionally experienced similar chest discomfort, although these episodes were less frequent and sometimes were associated with physical exertion.

Three years earlier, he was diagnosed with NSTEMI. At the time, his cardiac computed tomography coronary angiography (CTCA) revealed moderate stenosis in the right coronary artery (RCA) which was visually estimated, and mild stenosis in the left anterior descending artery (LAD). The patient was managed medically; however, he self-discharged prematurely to attend the funeral of his twin brother who had passed away after a sudden cardiac arrest. Further past medical history also included peripheral vascular disease and a previous ischaemic stroke which left him with little disabilities. The patient had a significant smoking history, averaging 20 cigarettes per day. He also had a positive family history of coronary artery disease.

Given his ongoing symptoms, he was referred again to cardiology. A stress echocardiogram Supplemental Video S1 and a 24 h ambulatory ECG were arranged to investigate further. The stress echocardiogram revealed no evidence of inducible ischaemia and indicated normal left ventricular function at peak stress.

**Figure 1:**
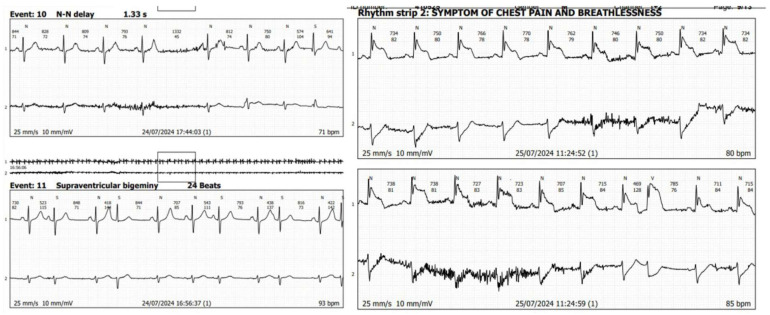
24-hour Holter showing transient ST elevation with associated chest pain and clamminess (**right panel**) and no ST elevation at baseline (**left panel**).

A 24 h Holter monitor Figure 1 showed an episode of ST elevation lasting six minutes, accompanied by his typical chest pain while walking at a normal pace, with associated clamminess, and shortness of breath.

Based on these findings, the patient was urgently called in for hospital admission and scheduled for an invasive coronary angiogram. His troponin levels were negative upon admission.

Upon arrival at the hospital, he reported crescendo symptoms at night and a few exertional episodes that were getting more frequent. He had no troponin rise and he was commenced on dual antiplatelet therapy for non-ST segment elevation acute coronary syndrome. Invasive angiogram was arranged for him which revealed moderate non-obstructive plaque disease in LAD and mid RCA which matched the computed tomography coronary angiogram (CTCA) from his previous admission. Intravascular ultrasound was done to rule out spontaneous dissection or plaque rupture events, with no such abnormalities seen. No intracoronary imaging was done.

At this point, we discussed with the patient the need for a microvascular study. His LAD and RCA were both assessed. His resting full-cycle ratio (RFR) to the RCA was negative at 0.93. Fractional flow reserve (FFR) of the RCA followed and was negative at 0.90, indicating no obstructive coronary artery disease to the RCA. His resting full-cycle ratio (RFR) to the LAD was negative at 0.94. Fractional flow reserve (FFR) of the LAD was negative at 0.92, indicating no obstructive coronary artery disease.

**Figure 2:**
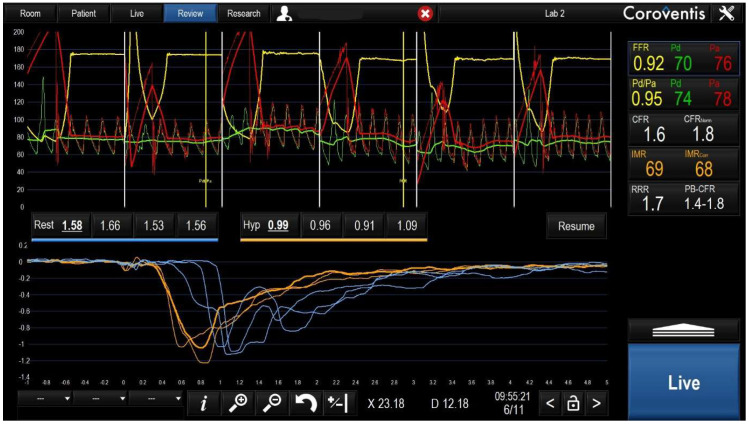
Microvascular study showing abnormal coronary flow reserve (CFR 1.6) and index of microcirculatory resistance (IMR 69).

A microvascular study of the LAD was then undertaken, confirming the diagnosis of microvascular dysfunction with a coronary flow reserve (CFR) of 1.6 (normal ≥ 2.5) and a significantly raised index of microcirculatory resistance (IMR) at 69 (normal ≤ 25) (Figure 2).

## 3. Coronary Artery Spasm (Vasospastic Angina)

**Figure 3:**
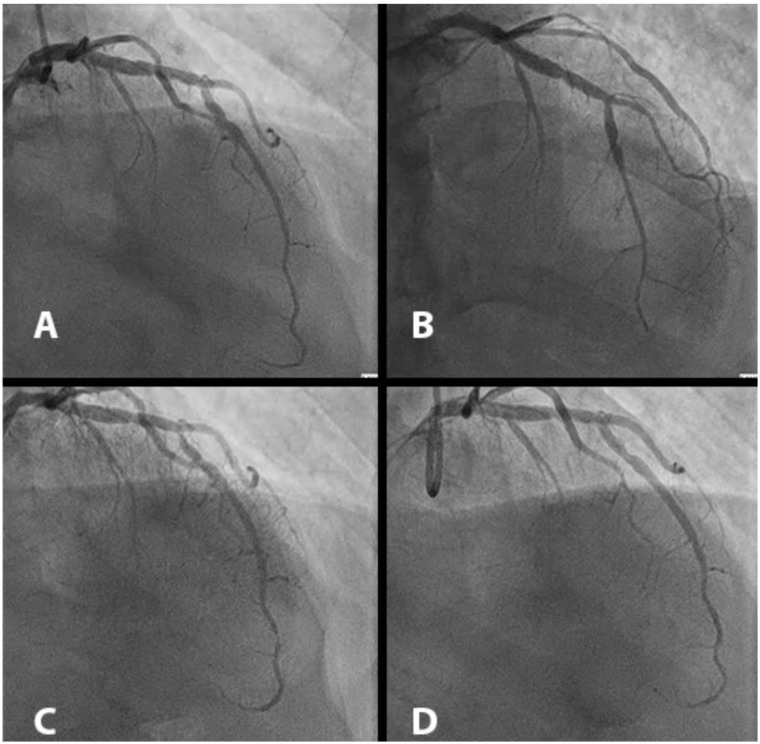
Wire-induced distal LAD coronary artery spasm. (**A**) Pre-wire. (**B**) Following wire removal after microvascular testing showing distal LAD occlusion. (**C**) Following 300 mcg of intracoronary nitrate showing severe vasoconstriction at site of occlusion but vessel now patent. (**D**) Following a further 300 mcg of intracoronary nitrate showing vessel normalization.

Following a microvascular assessment, coronary arteries can also be assessed for coronary artery spasm by giving sequential doses of acetylcholine to look for a vasoconstrictor response. This was not done in this case as following removal of the pressure wire, the patient developed chest pain and ST elevation. A coronary angiogram showed that the distal LAD was occluded. Following 300 mcg of intracoronary nitrate the occlusion improved, but the vessel remained with a significant stenosis at the occlusion point. Following a further 300 mcg of intracoronary nitrate, the vessel dilated back to normal, with the stenosis completely resolving (Figure 3).

This could have been followed by formal intracoronary acetylcholine testing to confirm inducible epicardial coronary spasm. However, we did not have this service in our centre at the time of the study. Wire-induced spasm supports the diagnosis given his presentation, prior symptoms and ECG changes on the Holter monitor; however, acetylcholine testing remains the reference-standard diagnostic test.

Given this patient’s nocturnal symptoms, together with a history of exertional chest pain, in combination with his microvascular and angiographic findings, his final diagnosis was that of a combination of both microvascular angina and spontaneous epicardial coronary artery spasm (combined microvascular and vasospastic angina).

Our findings fit with the current literature, with the CORMICA trial showing that when assessing patients with typical angina but no significant coronary artery disease on angiography, 72.2% had abnormalities on testing for microvascular dysfunction and/or coronary spasm in the catheter lab. The final diagnosis was microvascular angina in 51.7% of patients, vasospastic angina in 16.6% of patients and mixed microvascular and vasospastic angina in 20.5% of patients with INOCA, the diagnosis in our current case [4].

Prior to discharge, his bisoprolol was stopped; he was initiated on slow-release Diltiazem, and his slow-release Isosorbide Mononitrate was up-titrated. The rest of his medications include Aspirin, Atorvastatin, Lansoprazole, Ramipril, Ranolazine and Ticagrelor. He was also counselled on lifestyle modifications including smoking cessation.

This patient has been followed up as an outpatient in the clinic and via telephone call over a period of 6 months and he reported that the frequency and intensity of his symptoms were significantly reduced following the change in his medications, but that he still got occasional chest pain, especially at night. The patient also noted an increase in his exercise tolerance.

An understanding of the underlying mechanisms of INOCA is important not just as an academic exercise, but also in the case of our patient. While the primary function of the epicardial arteries is conducting blood across the heart, the microvasculature is primarily responsible for meeting the oxygen demands of the surrounding tissue [5]. This is done by increasing vasodilation and coronary blood flow. Coronary microvascular dysfunction occurs when the microvasculature is unable to meet the oxygen demand and adequately perfuse the myocardium under stressful conditions. In cases of microvascular spasm, this happens at rest [5]. This reduction in myocardial perfusion can be detected and measured as reduced coronary flow reserve (CFR) [3] which was also reduced in our patient.

In addition to the traditional risk factors of coronary artery disease such as atherosclerosis, hypertension, diabetes mellitus and dyslipidaemia that may or may not be present in patients with coronary microvascular dysfunction, patients with INOCA are also known to have additional risk factors such as inflammation. Patients with microvascular dysfunction have been documented to have higher C reactive protein levels than control subjects. They are also noted to have more ischaemic episodes on Holter monitoring [6]. They also have other risk factors such as depression and emotional stress which may contribute to the pathophysiology of the disease [7]. Our patient fits the bill with the loss of his twin brother who died suddenly due to a cardiac arrest.

Patients presenting with INOCA can be difficult to diagnose. The current case was that of a patient with both typical (exertional chest pain) and atypical (nocturnal rest pain) symptoms, with a normal stress echocardiogram and normal diagnostic coronary angiogram, ruling out epicardial coronary artery disease as the cause of his symptoms. Many such patients are told that they do not have a cardiac cause for their chest pain and are inappropriately discharged off cardiac medications, with their underlying cardiac condition remaining undiagnosed.

This has recently been recognised in the latest 2024 ESC Guidelines for the management of chronic coronary conditions [8]. These guidelines recognise that coronary microvascular dysfunction (CMD) is increasingly acknowledged as a prevalent factor characterising the entire spectrum of chronic coronary syndromes, and that functional and/or structural microcirculatory abnormalities may cause angina and ischaemia even in patients with non-obstructive disease of their epicardial coronary arteries (ANOCA and INOCA). When describing the recommended stepwise approach to the initial management of individuals with suspected chronic coronary syndrome, the guidelines state that “if symptoms persist after obstructive coronary artery disease is ruled out, coronary microvascular disease and vasospasm should be considered”. As a result, they recommend that guidewire-based CFR and/or microcirculatory resistance measurements should be considered in patients with persistent symptoms. In some patients, their coronary arteries are either angiographically normal or have moderate stenosis with preserved iFR/FFR measurements (Class IIa indication), as in the current case. They also recommend that intracoronary acetylcholine with ECG monitoring may be considered during angiography. Although acetylcholine was not given in the current case, spontaneous wire-induced coronary spasm was seen which reproduced symptoms and ST changes, responding to intracoronary nitrates, and given the clinical context, this was supportive of the diagnosis.

In this case, the patient was found to have abnormalities on his cardiac monitor (ST elevation in the presence of typical chest pain symptoms) and following referral for invasive microvascular assessment, was found to have abnormalities of microvascular function in the catheter lab (raised IMR, reduced CFR) as well as wire-induced spasm.

Coronary artery spasm leads to transient ischaemia and associated chest pain symptoms (Prinzmetal angina or vasospastic angina). Its transient nature can make diagnosis tricky as symptoms may present at rest and may have variable atypical triggers, often leading to a long delay in making the diagnosis, as in this case [9]. Vasospastic angina often occurs at night, demonstrating a circadian variation, potentially related to high vagal activity [10]. Functional testing with Dobutamine Stress Echo (DSE) or similar is often negative, as in this case [9]. If ambulatory ECG shows dynamic ischaemic changes in the presence of typical symptoms, then the diagnosis of coronary artery spasm is usually made [9]. His final diagnosis was INOCA due to both microvascular angina and vasospastic angina. Following diagnosis, appropriate treatment should be commenced, namely a vasodilating calcium antagonist and/or a slow-release nitrate, with beta-blocker therapy stopped. His treatment was altered accordingly, leading to a reduction in symptom burden following this change.

## 4. Learning Points

- Patients may present with atypical chest pain and this can often be misdiagnosed, and referred to the wrong specialty, leading to wrong management.
- When chest pain due to ischaemia is investigated, the cause may not be found even with invasive angiography and computer tomography coronary angiogram.
- In patients with persistent symptoms, the need for a microvascular study should be assessed as this may reveal microvascular angina and possible coronary artery spasms which may explain the patient’s symptoms and guide further management.

## 5. Conclusions

INOCA is a common cardiological finding and as we have seen in this case, their presentation and investigation findings could be unexpected. Despite negative coronary angiography, a high index of suspicion is necessary and invasive coronary functional testing is important in selected patients with persistent angina and non-obstructive coronary arteries to establish the diagnosis. Once diagnosis is established, appropriate management and follow-up would be required to control their symptoms and improve their quality of life.

NB: Funding is provided by the authors and their institutions, with no conflict reported.

Informed consent was obtained from the patient prior to writing this case report.

## Data Availability

The original contributions presented in this study are included in the article/Supplementary Materials. Further inquiries can be directed to the corresponding author.

## Supplementary Materials

The following supporting information can be downloaded at: https://www.mdpi.com/article/10.3390/reports9030196/s1.

## Abbreviations

**CFR**: Coronary flow reserve is a metric used to determine how much increase in blood flow the heart can give under stress (e.g., exercise) than at rest. **IMR:** Index of microvascular resistance reflects the resistance of the coronary microcirculation during peak blood flow. **RFR:** Resting full-cycle ratio is used to measure the functional severity of coronary artery stenoses. **FFR:** Fractional flow reserve measures pressure and blood flow in narrowed coronary arteries. It helps to determine if the stenoses or blockage of said coronary artery is severe enough to cause ischaemia and would thus require stenting.
